# Protease-Sensitive Synthetic Prions

**DOI:** 10.1371/journal.ppat.1000736

**Published:** 2010-01-22

**Authors:** David W. Colby, Rachel Wain, Ilia V. Baskakov, Giuseppe Legname, Christina G. Palmer, Hoang-Oanh B. Nguyen, Azucena Lemus, Fred E. Cohen, Stephen J. DeArmond, Stanley B. Prusiner

**Affiliations:** 1 Institute for Neurodegenerative Diseases, University of California, San Francisco, California, United States of America; 2 Department of Neurology, University of California, San Francisco, California, United States of America; 3 Department of Pathology, University of California, San Francisco, California, United States of America; 4 Department of Cellular and Molecular Pharmacology, University of California, San Francisco, California, United States of America; University of Edinburgh, United Kingdom

## Abstract

Prions arise when the cellular prion protein (PrP^C^) undergoes a self-propagating conformational change; the resulting infectious conformer is designated PrP^Sc^. Frequently, PrP^Sc^ is protease-resistant but protease-sensitive (s) prions have been isolated in humans and other animals. We report here that protease-sensitive, synthetic prions were generated *in vitro* during polymerization of recombinant (rec) PrP into amyloid fibers. In 22 independent experiments, recPrP amyloid preparations, but not recPrP monomers or oligomers, transmitted disease to transgenic mice (*n* = 164), denoted Tg9949 mice, that overexpress N-terminally truncated PrP. Tg9949 control mice (*n* = 174) did not spontaneously generate prions although they were prone to late-onset spontaneous neurological dysfunction. When synthetic prion isolates from infected Tg9949 mice were serially transmitted in the same line of mice, they exhibited sPrP^Sc^ and caused neurodegeneration. Interestingly, these protease-sensitive prions did not shorten the life span of Tg9949 mice despite causing extensive neurodegeneration. We inoculated three synthetic prion isolates into Tg4053 mice that overexpress full-length PrP; Tg4053 mice are not prone to developing spontaneous neurological dysfunction. The synthetic prion isolates caused disease in 600–750 days in Tg4053 mice, which exhibited sPrP^Sc^. These novel synthetic prions demonstrate that conformational changes in wild-type PrP can produce mouse prions composed exclusively of sPrP^Sc^.

## Author Summary

Prions are infectious proteins that cause heritable, sporadic, and transmissible diseases in humans and other mammals. These infectious proteins arise when the normal form of the prion protein (PrP) adopts a self-perpetuating conformation. This disease-causing PrP form is frequently distinguished from normal PrP by its resistance to digestion by proteases although considerable evidence shows that protease-sensitive prions occur naturally in humans and sheep. Here we describe the generation of novel protease-sensitive synthetic prions. After producing recombinant PrP of the wild-type mouse sequence in *Escherichia coli*, we polymerized the protein into an amyloid fiber conformation. Mice inoculated with these amyloid fibers developed extensive neurodegeneration characteristic of prion disease, but did not generate protease-resistant PrP. Prions from sick animals were transmitted to healthy animals, which likewise developed neurodegeneration but not protease-resistant prions. These novel synthetic prions demonstrate that truncated wild-type PrP can undergo a conformational change that becomes infectious yet the protein remains protease sensitive.

## Introduction

Prions are infectious proteins that cause heritable, sporadic, and transmissible disease in humans and other mammals [1]. The molecular basis of prion disease is a conformational change in the normal, cellular prion protein, denoted PrP^C^, to a disease-causing form, denoted PrP^Sc^ [2],[3]. This conformational change has often been detected by measuring the extent to which PrP resists digestion by proteases, such as proteinase K (PK), because most naturally occurring prion strains are partially resistant to digestion [4],[5],[6],[7]. However, a substantial portion of some prion strains is comprised of protease-sensitive (s) PrP^Sc^; for example, over 90% of PrP^Sc^ in the brains of some sporadic Creutzfeldt-Jakob disease (sCJD) cases is sensitive to PK digestion [8]. Importantly, cases of fatal neurological disease have been reported with neuropathology typical of sCJD but harboring no protease-resistant (r) PrP^Sc^ [9],[10], and the PrP(H187R) mutation gives rise to neurological disease with an abnormal PrP conformer that is sensitive to protease digestion [11]. Atypical strains causing scrapie, a prion disease in sheep, have also been reported with a high proportion of sPrP^Sc^ [12],[13],[14].

Transgenic (Tg) mice expressing mouse (Mo) PrP with the P101L mutation corresponding to the human P→L mutation causing Gerstmann-Sträussler-Scheinker (GSS) disease also harbor protease-sensitive prions. Tg(PrP,P101L) mice expressing high levels of mutant PrP spontaneously develop prion disease and generate a mutant form of PrP^Sc^ that is resistant only to mild PK digestion [15],[16],[17]. Tg(PrP,P101L)196 mice expressing low levels of mutant PrP were inoculated with brain extracts from ill Tg mice overexpressing mutant PrP or a synthetic, 55-residue PrP(P101L) peptide refolded into a β-rich conformation [18],[19]. In the inoculated Tg196 mice, both the brain extracts and the synthetic peptide hastened the development of neurodegenration [15],[16],[20]. Interestingly, prions with the P101L mutation were not transmissible to mice expressing the wild-type (wt) PrP sequence; whether this was due to the protease sensitivity of the prions or the presence of the P101L mutation was not clear.

Inoculation of seeded and unseeded preparations of recMoPrP(89–230) amyloid fibers into Tg9949 mice, which express a similar, N-terminally truncated PrP at 16–32× the levels of PrP in Syrian hamster brain [21], generated prions [22]. The brains of mice that had been inoculated with the seeded PrP amyloids produced a synthetic prion strain denoted MoSP1, which exhibited protease resistance and shortened incubation periods upon serial passage to both wt and Tg lines of mice [22],[23],[24]. The extent to which the brains of mice that had been inoculated with unseeded fibers harbored protease-resistant PrP was unclear [22]. We hypothesized that Tg9949 mice inoculated with the unseeded amyloid fibers, as described in our initial report [22], may contain protease-sensitive prions since their brains exhibited all the neuropathological features of prion disease. At that time, the most reliable method of detecting sPrP^Sc^ was the conformation-dependent immunoassay (CDI) [7],[25], which consists of selective precipitation of PrP^Sc^ by phosphotungstate (PTA) followed by immunodetection. However, the CDI proved unreliable in detecting sPrP^Sc^ due to the high levels of the transgene product N-terminally truncated PrP^C^. For this reason, we sought an alternative method for detecting sPrP^Sc^; we called this new procedure the amyloid seeding assay (ASA). The ASA employs PTA precipitation, similar to the CDI, but detects prions based on their propensity to hasten the formation of PrP amyloids. We found that prions could be detected using the ASA in brain samples from Tg9949 mice inoculated with the unseeded fibers [26].

Recently, several new strains of protease-resistant synthetic prions have been created from amyloid generated under a variety of conditions and inoculated into mice that overexpress full-length PrP [27]. These findings expand the original report of synthetic prions [22] to a second line of transgenic mice and confirm the ability to create protease-resistant synthetic prions.

To extend our discovery that truncated wt mammalian prions could be produced synthetically [22],[27], we performed a large series of experiments with various recMoPrP amyloid fibers in Tg9949 mice. We sought conditions to produce synthetic prions with abbreviated incubation times. While we investigated numerous variations in the preparation of recMoPrP amyloids, none resulted in a shortening of the incubation times. However, most of the amyloid preparations caused prion disease in Tg9949 mice as demonstrated by neuropathological changes and the presence of sPrP^Sc^. These protease-sensitive prions transmitted disease to two different Tg lines of mice. Unexpectedly, control, uninoculated and mock-inoculated Tg9949 mice were prone to late-onset neurological dysfunction that was indistinguishable clinically from Tg mice inoculated with protease-sensitive prions. But the ill, control Tg9949 mice did not develop neurodegeneration, form sPrP^Sc^ or transmit prion disease.

The studies reported here not only demonstrate the validity of the experimental systems reported earlier but they also extend our understanding of synthetic prions. Moreover, our findings establish that wt sPrP^Sc^ alone, in the absence of detectable rPrP^Sc^, is sufficient to cause neurodegeneration.

## Results

### Control Tg9949 mice develop spontaneous neurological dysfunction

**Figure 1:**
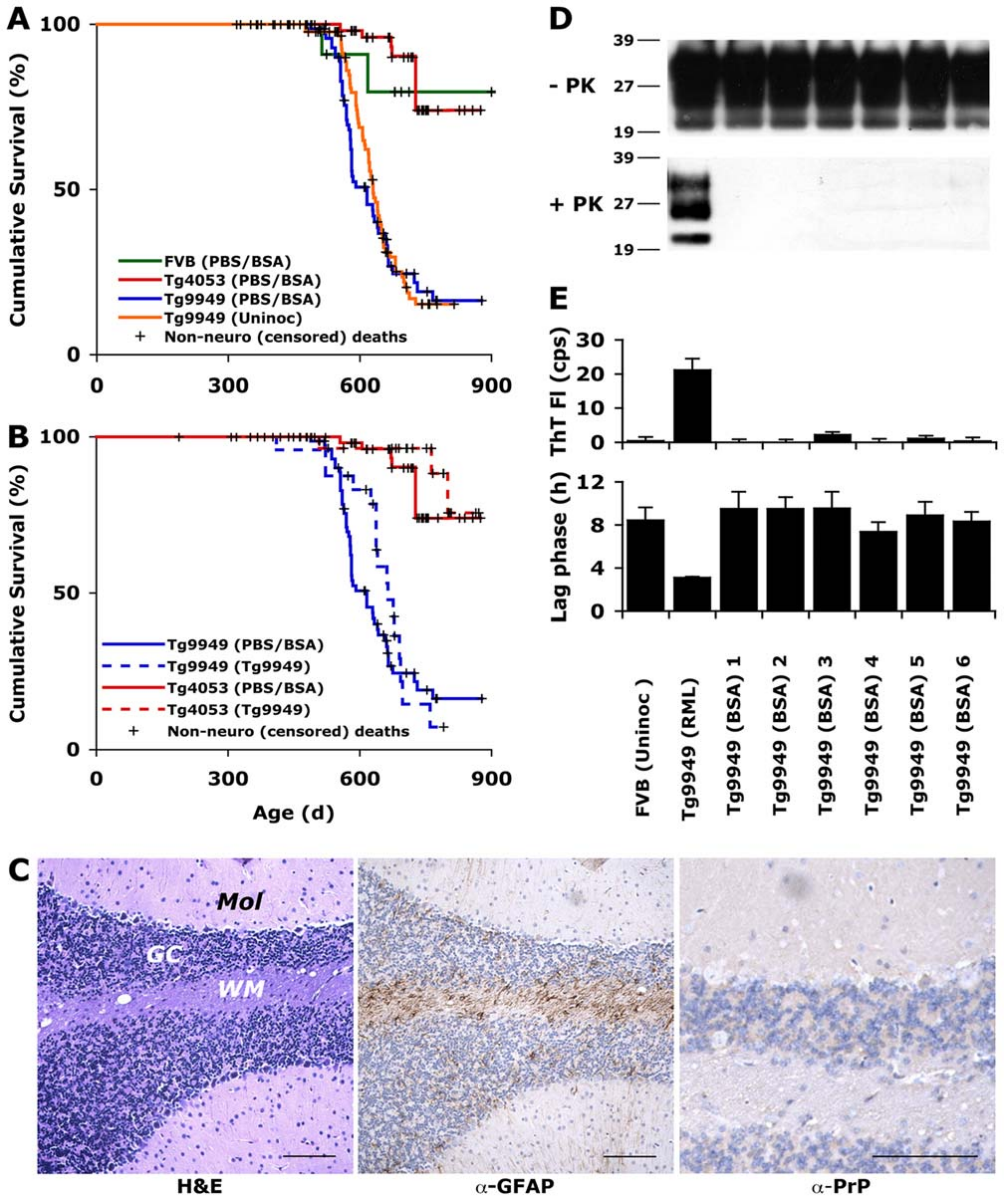
Tg9949 mice are prone to neurological dysfunction but do not spontaneously generate prions. (**A**) Wild-type (FVB), Tg4053, and Tg9949 mice were monitored for signs of neurological dysfunction for >900 days. Both uninoculated and BSA-inoculated Tg9949 mice were more likely than wt mice (p = 0.03) and Tg4053 mice (p<0.001) to develop neurological dysfunction. However, prions could not be detected in the brains of neurologically impaired Tg9949 mice, as determined by bioassay in Tg9949 and Tg4053 mice (**B**), by histopathological staining (**C**), by Western immunoblotting (**D**), and by the amyloid seeding assay (**E**). (**C**) Brain sections of neurologically impaired Tg9949 mice were stained with H&E to visualize vacuoles (left), α-GFAP to visualized astrocytic gliosis (middle), and α-PrP to visualize PrP^Sc^ deposits (right). Scale bars represent 100 µm. Mol, molecular cell layer; GC, granular cell layer; WM, white matter. Additional neuropathological analyses of control brains are shown in **Fig. S1**. (**D**) Western immunoblots of undigested and PK-digested brain samples from six aged Tg9949 mice show no rPrP^Sc^. Homogenate from a Tg9949 mouse inoculated with RML prions is shown as a positive control. Lane assignments are as indicated in panel E. (**E**) Brain samples from six aged Tg9949 mice do not seed the formation of recPrP amyloid, as judged by an increase in Thioflavin T fluorescence (top) and by a decrease in the lag phase for amyloid formation (bottom). An uninoculated FVB mouse brain is included as a negative control. Negative controls in (A) are pooled results, including some previously published data [27].

To determine if Tg9949 mice generate prions spontaneously, 96 uninoculated Tg9949 mice and 78 Tg9949 mice inoculated with bovine serum albumin (BSA) were monitored twice weekly for signs of neurological dysfunction. We found that a cumulative incidence of 85% of these control Tg mice developed late-onset ataxia at approximately 600 d (**Fig. 1A** and **Table S1**). The most common clinical observations of aged Tg9949 mice were ataxia, circling, and a dull coat. Mice inoculated with BSA were no more likely than uninoculated mice to develop neurological dysfunction (**Fig. 1A** and **Table S1**). We compared the probability of these Tg9949 mice developing ataxia in old age to the probability that other Tg and wt mice develop ataxia. We found that Tg9949 mice are significantly more likely to develop ataxia than wt FVB mice (*n* = 12; p = 0.03) and Tg mice that express full-length PrP at 4–8 times wt levels (Tg4053 mice, *n* = 62; p<0.001) [17],[27],[28]. Older Tg4053 and FVB mice had comparable rates (p>0.30) of neurological dysfunction.

We used four different methods to determine if Tg9949 mice suffering from neurological dysfunction had spontaneously generated prions: bioassay, neuropathology, Western blotting for rPrP^Sc^, and ASA for sPrP^Sc^ [26]. For bioassays, brains from three Tg9949 mice exhibiting neurological dysfunction were homogenized and inoculated intracerebrally (ic) into weanling Tg9949 and Tg4053 mice. Inoculation of these brain homogenates neither hastened the onset of neurological dysfunction in Tg9949 mice nor resulted in neurological dysfunction in Tg4053 mice (**Fig. 1B**). In contrast, inoculation of Rocky Mountain Laboratory (RML) prions into Tg9949 and Tg4053 mice mice resulted in disease in 161 d and 50 d, respectively [21].

For neuropathological analyses, we examined more than 20 brain samples from Tg9949 mice exhibiting neurological dysfunction (from both the uninoculated and BSA-inoculated groups; **Fig. 1A**). Typically, neuropathologic features of prion disease include the formation of vacuoles, proliferation of astrocytes, and deposition of PrP aggregates [29]. We found no evidence of prion disease pathology in any of the brains taken from aged Tg9949 mice (a representative specimen is shown in **Fig. 1C**). Occasional vacuoles and mild astrocytic gliosis of the cerebellar white matter were observed, but these findings were consistent with aging (for comparison with aged, healthy Tg9949, wt, and Tg4053 mice, see **Fig. S1**). Neuropathological analysis did not indicate the cause of neurological dysfunction in older uninoculated or BSA-inoculated Tg9949 mice.

To determine whether Tg9949 mice suffering from neurological dysfunction harbored protease-resistant PrP, we performed Western immunoblotting of brain samples. In over 100 mouse brains from uninoculated and BSA-inoculated Tg9949 mice, we found no PK-resistant PrP signal. Six independent samples are shown in **Fig. 1D**.

Next, we subjected the brain homogenates of Tg9949 mice to the ASA (**Fig. 1E**) [26]. This assay is based on the observation that prions, partially purified from brain homogenates by PTA precipitation [7], accelerate the conversion of recPrP into a conformation that favors amyloid assembly [26]. We incubated PTA-precipitated brain homogenates with recMoPrP(89–230) for 6 h and monitored amyloid formation by measuring the fluorescence emission of Thioflavin T (ThT) [30]. As depicted, samples from RML prion-inoculated animals were active in the ASA whereas samples from BSA-inoculated Tg9949 mice were not (**Fig. 1E**, top). Because amyloid seeding is a kinetic process, we wanted to be certain that none of the samples had an intermediate effect on amyloid formation that did not register on the time scale of the initial measurement (6 h). We measured the mean lag phase for amyloid formation for all of the samples, and found that all uninoculated and BSA-inoculated Tg9949 samples showed lag times similar to uninoculated FVB control brains (**Fig. 1E**), indicating that aged Tg9949 mice do not spontaneously form protease-sensitive prions. In contrast, the brains of RML prion-inoculated mice were able to reduce the lag phase for amyloid formation (**Fig. 1E**, bottom).

### Inoculation of Tg9949 mice with monomeric, oligomeric and amyloid PrP

We inoculated Tg9949 mice with recPrP(89–230) in α-helical (monomeric), β-rich oligomeric and amyloid forms. In addition to the two amyloid inoculations previously reported [22] (Amyloids 1 and 2; **Table S2**), we made 24 independent amyloid preparations by systematically varying the conditions used for amyloid formation including: (1) the initial conformation of recMoPrP, (2) the composition and concentration of denaturant, (3) the number of times the seeding procedure was repeated, (4) use of multiple freeze-thaw cycles, and (5) the method used to purify the fibers prior to inoculation (Amyloids 3–4, Amyloids 14–35, **Table S2**). We inoculated monomeric recMoPrP(89–230), oligomeric recMoPrP(89–230), and each of the 24 new amyloid preparations into groups of at least eight Tg9949 mice. All inoculated Tg9949 mice developed neurological dysfunction between 500 and 650 days (**Table S3**). Tg9949 mice inoculated with protease-sensitive synthetic prions had clinical presentations that were indistinguishable from control mice as they aged, specifically, ataxia, circling, and a dull coat.

**Figure 2:**
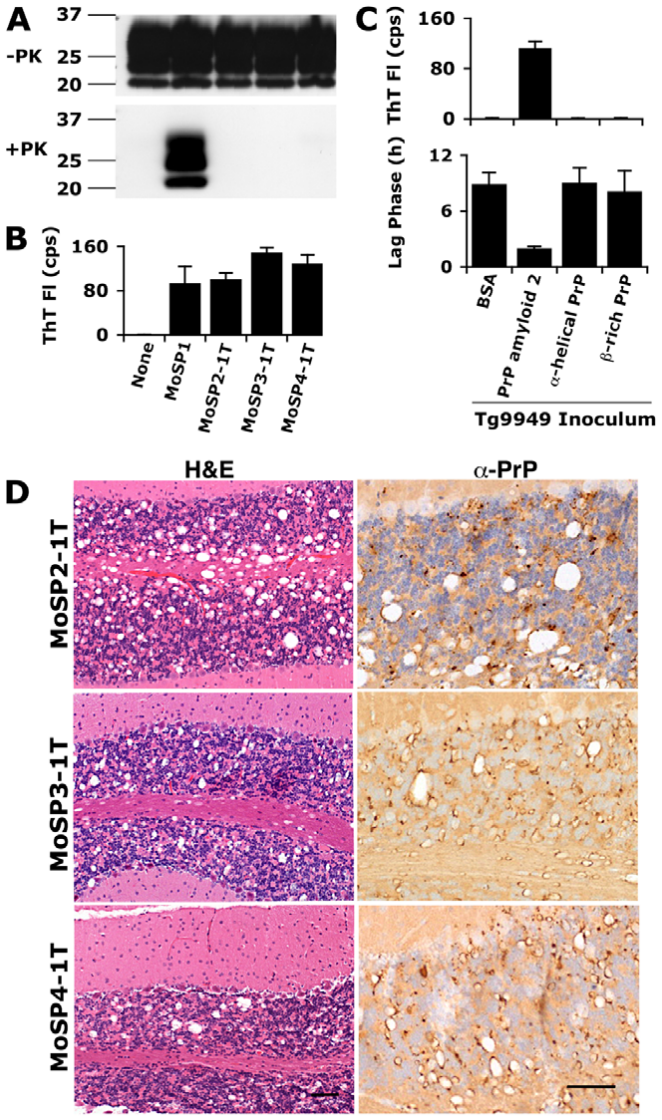
Inoculation of Tg9949 mice with PrP amyloid, but not other PrP conformations, results in the generation of prions. Tg9949 mice were inoculated with recPrP folded in various conformations (α-helical, β-rich, and amyloid) and allowed to live out their normal life span (**Table S2** and **S3**). Tg9949 mice inoculated with BSA or MoSP1 [22] are shown as controls. The first transmission (1T) of MoSP2, MoSP3, and MoSP4 refers to inoculation of Tg9949 mice with amyloid 2, amyloid 3, and amyloid 4 preparations, respectively. Brain samples from Tg9949 mice containing MoSP2, MoSP3, or MoSP4 did not harbor protease-resistant PrP (**A**) but showed activity in the ASA (**B**; kinetic data shown in **Fig. S2**; note that the sample amount for ASA is 1/1000 of the amount used in Western blots). Brain samples from mice inoculated with non-amyloid PrP conformations did not show activity in the ASA (**C**). Activity in the ASA was detected by an increase in ThT fluorescence (**B and top of panel C**) and by a decrease in the mean lag phase for PrP amyloid formation (**bottom of panel C**). (**D**) Brain sections of Tg9949 mice containing MoSP2, MoSP3, or MoSP4 showed neuropathology consistent with prion disease, including extensive vacuolation (H&E stain, left column) and PrP deposition (anti-PrP stain, right column). Each scale bar represents 100 µm and applies to the micrographs in the same column.

To determine if the brains of inoculated Tg9949 mice harbored prions, we analyzed brain samples by Western immunoblotting and the ASA (**Fig. 2A–C** and **Table S3**). In the brains of mice that had been inoculated with Amyloids 2, 3, or 4, no PK-resistant PrP was detected using any of three different antibodies (P, D18, and R2, which bind to the N-terminal, middle, and C-terminal regions of PrP(89–230), respectively; immunoblot probed with P is shown in **Fig. 2A**). Likewise, no PK-resistant PrP was detected in the brains of mice inoculated with Amyloids 14–35 by immunoblotting with the antibody D18 (**Table S3**). However, brain samples from mice that had been inoculated with 21 of the 24 new amyloid preparations showed substantial activity in the ASA, indicating the presence of prions; for the remaining three amyloids, no prions were detected (**Figs. 2B** **, S2**, and **Table S3**). We found that the brains of Tg9949 mice inoculated with PrP in an α-helical, monomeric conformation [31] and those inoculated with PrP in a β-rich oligomeric form [32] did not contain PrP in a conformation that was active in the ASA (**Fig. 2C**, top). We also measured mean lag phases in the ASA to be certain that no intermediate seeding effect had occurred (**Fig. 2C**, bottom). Examination of the brains of ill, amyloid-inoculated animals by histopathology revealed the hallmarks of prion disease, including extensive vacuole formation and PrP deposits, either lining the vacuoles or as punctate aggregates near the vacuoles (**Fig. 2D** and **Table S3**). Tg9949 mice inoculated with the α-helical or β-oligomeric recPrP had normal brains histologically with no evidence of prion disease. Based on the ASA activity and neuropathology, we conclude that 21 of the 24 new amyloid preparations resulted in the formation of protease-sensitive prions, which were transmissible to Tg9949 mice. We chose three brain isolates for further study and designated the resulting prion strains MoSP2, MoSP3, and MoSP4, respectively.

Because this was the first time that the ASA has been applied to a large number of unknown samples, we analyzed the correlation of this method to neuropathological analysis. Forty-six samples were analyzed for the presence of prions both by neuropathology and the ASA; of these, 34 were positive by both methods, 11 were negative in both, and 1 was positive in the ASA but negative by neuropathology (**Table S4**). Thus, results by the ASA correlated with neuropathologic assessment for 98% of samples (p<0.001).

### Serial transmission of protease-sensitive synthetic prions in Tg9949 mice

Brain homogenates from ill Tg9949 mice containing MoSP2, MoSP3, and MoSP4 prions were inoculated ic into Tg9949 mice. Brain homogenates from aged Tg9949 mice with neurological dysfunction were used as controls. Serial transmission (or second transmission, 2T) of all three protease-sensitive synthetic prion strains in Tg9949 mice resulted in neurological dysfunction within a timeframe comparable to uninoculated, control mice (**Table S5**). A third transmission (3T) of MoSP2 into Tg9949 mice gave similar results (**Table S5**).

**Figure 3:**
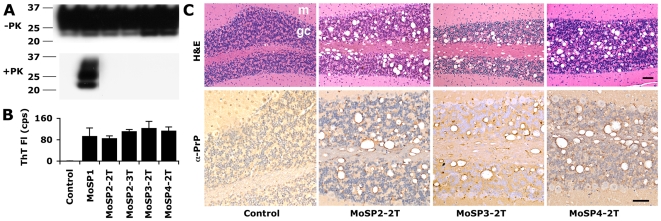
Protease-sensitive synthetic prions are serially transmissible in Tg9949 mice. MoSP2-1T, MoSP3-1T and MoSP4-1T were serially passaged to new groups of Tg9949 mice for a second transmission (2T) by intracerebral inoculation of brain homogenate containing each isolate. MoSP2-2T was serially passaged an additional time for a third transmission (3T). In each case, the animals lived a normal life span (**Table S4**). The brains of Tg9949 mice containing MoSP2-2T, MoSP3-2T, or MoSP4-2T showed no protease-resistant PrP by Western blotting (**A**), but activity in the ASA (**B**) and neuropathology consistent with prion disease (**C**). Brain samples from mice inoculated either with MoSP1 [22] or with homogenates of uninfected Tg9949 mice are shown as controls. (**C**) Cerebellar sections were stained with H&E (top row) and α-PrP (bottom row). m, molecular layer; gc, granule cell layer. Each scale bar represents 100 µm and applies to the panels in the same row.

We wished to determine whether the protease sensitivity and ASA activity of MoSP2, MoSP3, and MoSP4 were maintained upon serial passage in Tg9949 mice. Western blots of brain samples from Tg9949 mice serially infected with MoSP2, MoSP3, and MoSP4 were probed with anti-PrP antibody P and revealed no protease-resistant PrP fragments (**Fig. 3A**). MoSP1 was used as a PK-resistant positive control. Employing lower concentrations of PK (1, 3, and 10 µg/ml) revealed no difference between Tg9949 mice inoculated with MoSP2 and uninoculated Tg9949 controls (**Fig. S3**). We next subjected brain homogenates of mice that had received serial transmission of MoSP2, MoSP3, and MoSP4 to the ASA. PTA-purified brain homogenates were incubated with recMoPrP(89–230) for 6 h, and amyloid formation was monitored by ThT fluorescence. MoSP2, MoSP3, and MoSP4 serially passaged in Tg9949 mice exhibited consistent activity in the ASA (**Fig. 3B**). In contrast, brain homogenates from control mice inoculated with a mock inoculum (Tg9949 brain homogenate) did not seed amyloid formation.

Next, we analyzed brain sections of Tg9949 mice serially infected with MoSP2, MoSP3, and MoSP4. Serial passage of each protease-sensitive synthetic prion strain resulted in substantial vacuolation and formation of PrP deposits (**Fig. 3C**). Vacuolation scores, or the area of a region occupied by vacuoles, were tabulated for various brain regions from the initial transmission, second transmission, and third transmission of MoSP2 in Tg9949 mice (**Fig. S4**). Vacuolation in Tg9949 mice infected with MoSP2 by serial passage was similar to that in Tg9949 mice originally inoculated with amyloid fibers, indicating that the strain characteristics of MoSP2 were conserved upon passage. Finally, brain sections of mice inoculated with MoSP2 were subjected to histoblot analysis with and without PK digestion (**Fig. S5**), which confirmed that PrP deposits in the brains of mice inoculated with MoSP2 are protease-sensitive.

### Serial transmission of protease-sensitive synthetic prions to mice expressing full-length PrP

**Figure 4:**
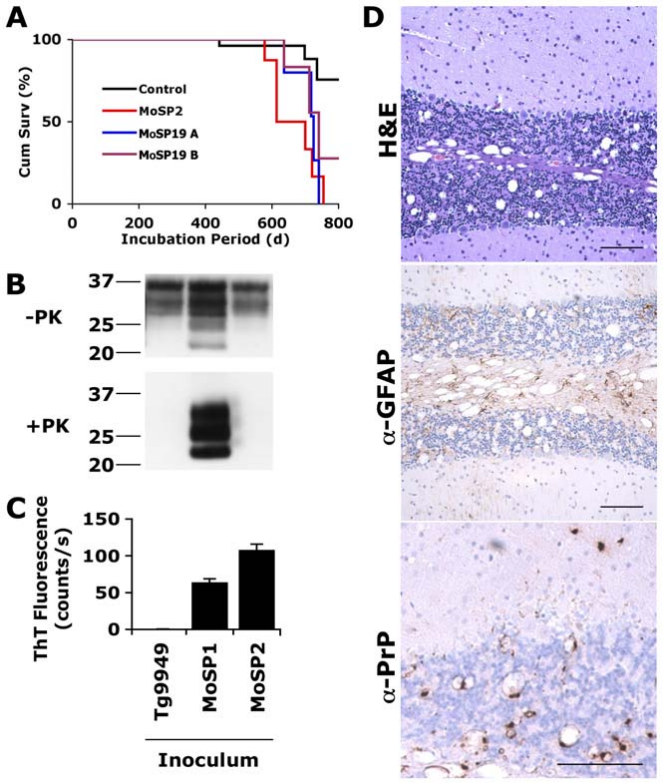
Protease-sensitive synthetic prions are serially transmissible to Tg4053 mice. Tg4053 mice intracerebrally inoculated with MoSP2-1T (red), MoSP19A (blue) or MoSP19B (purple) developed signs of prion disease between 600–750 d (**A**). MoSP19A and MoSP19B are two Tg9949 brain isolates inoculated with Amyloid Prep 19 (origin in **Table S3**). Tg4053 mice inoculated with protease-sensitive synthetic prions were significantly more likely to develop neurological dysfunction than mice inoculated with uninfected Tg9949 brain homogenate (black, p<0.001). PrP in the brains of these mice was sensitive to PK digestion (**B**) and active in the ASA (**C**). MoSP1 was used as a control. The brains of ill, MoSP2-inoculated Tg4053 mice showed neuropathology consistent with prion disease (**D**), including vacuolation (top panel), astrocytic gliosis (middle panel), and punctate PrP deposits (bottom panel). Scale bars represent 100 µm.

Tg9949 brain homogenates containing MoSP2 were inoculated ic into Tg4053 mice, which overexpress full-length MoPrP-A. Additionally, two Tg9949 brain homogenates inoculated with Amyloid Prep 19 (**Table S3**) were passaged to Tg4053 mice. In contrast to Tg9949 mice, Tg4053 mice are not prone to developing late-onset ataxia (**Fig. 1A**). Transmission of MoSP2 and the other protease-sensitive synthetic prion isolates to Tg4053 mice resulted in prion disease with incubation periods of 600–750 d (**Fig. 4A**). Tg4053 mice inoculated with protease-sensitive synthetic prion isolates were significantly more likely to develop neurological dysfunction than Tg4053 mice inoculated with brain homogenate from uninoculated aged Tg9949 mice (p<0.001). Brain samples of Tg4053 mice inoculated with protease-sensitive synthetic prion isolates showed no rPrP^Sc^ in Western blots (MoSP2 shown in **Fig. 4B**), but substantial activity in the ASA (MoSP2 shown in **Fig. 4C**). In contrast, Tg4053 mice inoculated with control Tg9949 brain homogenates had neither rPrP^Sc^ nor sPrP^Sc^. To detect trace quantities of rPrP^Sc^ in Tg4053 mice inoculated with MoSP2, we subjected 1 ml of 5% brain homogenate to PK digestion (20 µg/ml), followed by PTA precipitation (**Fig. S6**). The PTA pellet was resuspended in 100 µl of 10% SDS and boiled. Thirty microliters of the resulting product was then analyzed by Western immunoblotting, approximately 10-fold more material than used elsewhere in this work for Western blots and 1000-fold more material than used for the ASA. Even under these conditions, no rPrP^Sc^ could be detected. Neuropathology consistent with prion disease was observed in brain sections from MoSP2-inoculated Tg4053 mice (**Fig. 4D**). Punctate PrP deposits and vacuolation were widespread, but most severe in the CA1 region of the hippocampus and in the cerebellum (**Fig. S4**). From these data, we conclude that protease-sensitive synthetic prions in the brains of Tg9949 mice were transmitted to Tg4053 mice, and the resulting prions were composed of sPrP^Sc^ and produced neuropathologic changes typical of prion disease. Notably, MoSP2 produced no clinical or pathologic evidence of prion disease in wt FVB mice (**Table S6**).

## Discussion

Encouraged by the production of prion infectivity by polymerizing recMoPrP(89–230) into amyloid fibers [22],[23], we undertook a study aimed at identifying conditions that would shorten incubation times for synthetic prions in Tg mice. We explored an array of variables, including the composition and concentration of denaturant, the number of seeding rounds, and the number of freeze-thaw cycles, none of which modified experimental outcomes. Twenty-five preparations of recMoPrP(89–230) polymerized into amyloid were inoculated into 204 Tg9949 mice. Eighty percent (or 164) of the Tg9949 mice were found to have sPrP^Sc^ and neuropathology typical of experimental prion disease. Three of the amyloid preparations failed to produce measurable sPrP^Sc^ and neuropathology while six other preparations showed incomplete transmissions (**Table S3**). Three of the 22 infectious, recMoPrP amyloid preparations were studied in detail; these were designated MoSP2, MoPSP3 and MoSP4. Each of these synthetic prion isolates transmitted disease upon serial passage in Tg9949 mice (**Fig. 3**). In addition, MoSP2 and two other protease-sensitive synthetic prion isolates transmitted disease to Tg4053 mice overexpressing MoPrP (**Fig. 4**).

Our creation of these novel protease-sensitive prions challenges the accepted definition of what constitutes a prion. Mammalian prions have been most closely associated with PrP that resists protease digestion [4],[5],[6],[7]. Additionally, mammalian prions typically cause disease that shortens the lifespan of the animal. While the novel synthetic prions reported here do not have either of these characteristics, they share four traits common to all mammalian prions: (1) they possess an alternatively folded isoform of PrP (**Fig. 2B**); (2) they cause neurologic dysfunction in animals (**Fig. 4A**); (3) they cause profound neuropathologic changes (**Fig. 2D** **and** **4D**); and (4) they are transmissible (**Figs. 3** **and** **4**). We suggest that these four traits define mammalian prions.

Many prions observed in nature appear to be composed of mixtures of rPrP^Sc^ and sPrP^Sc^ [7],[8],[12],[13],[14],[25], though the relationship between the two is unclear. The creation of synthetic prions composed solely of sPrP^Sc^ offers new insight into this relationship and the role of sPrP^Sc^ in disease. Our results demonstrate that sPrP^Sc^ is transmissible and causes neurodegeneration in the absence of rPrP^Sc^. Our findings also suggest that sPrP^Sc^ does not arise as an off-pathway product during the replication of rPrP^Sc^. Examples of natural prion diseases that feature sPrP^Sc^ predominantly are rarely reported [9],[10]. In the work reported here, it was necessary to use genetically modified lines of mice to make this unusual prion phenotype more readily accessible. Notably, inoculation of wt FVB mice with the amyloid fibers used in these studies did not result in prion disease (**Table S7**).

It is intriguing that MoSP2 remained protease-sensitive even after repeated serial passage. The protease-sensitive prion fraction isolated from Syrian hamsters infected with 263K prions was shown to give rise to rPrP^Sc^ in the protein misfolding cyclic amplification assay [33]. Our findings indicate that infection with sPrP^Sc^ does not necessarily lead to rPrP^Sc^ generation.

Because some lines of Tg mice overexpressing wt PrP develop spontaneous neurological dysfunction [34], we observed 96 uninoculated, control Tg9949 mice and ic inoculated 78 control Tg9949 mice with BSA in PBS. Unexpectedly, most of these control Tg9949 mice developed late-onset, spontaneous neurological dysfunction. All the ill, control Tg9949 mice showed no neuropathological changes typical of prion disease. Additionally, no sPrP^Sc^ or rPrP^Sc^ was detected in the brains of these control Tg9949 mice. These studies established the validity and limitations of transmitting prions to Tg9949 mice.

In our initial report of synthetic prions, we described the onset of neurological dysfunction in Tg9949 mice between 380 and 660 days after inoculation [22]. Three sets of Tg9949 mice were used as controls. In the first set, 10 of 12 healthy, uninoculated Tg mice were terminated at 574 days of age; the other two Tg9949 mice developed signs of neurological dysfunction at 564 and 576 days of age but had neither rPrP^Sc^ nor neuropathology typical of prion disease. In the second set of control mice, eight Tg9949 mice were inoculated with Syrian hamster Sc237 prions and were healthy at 525 days of age when they were sacrificed. Third, seven Tg9949 mice were inoculated with PBS and remained healthy at 672 days of age when they were sacrificed. In light of the current work, the first and second control groups were terminated too early to observe neurological dysfunction and the third group appears to be an outlier. Our discovery that Tg9949 mice develop late-onset neurological dysfunction does not undermine the key finding of the earlier work [22], which demonstrated that prions could be generated *de novo* from recombinant protein, but it does raise the possibility that the incubation period for the initial transmission may have been longer than reported. Incubation periods for some prion strains in Tg9949 mice cannot be determined when they approach or exceed the age of onset of spontaneous neurological dysfunction in these mice.

Despite the observation that uninoculated, control Tg9949 mice were prone to ataxia in old age, we found no evidence of prions in these mice by biochemical means, by histopathology, or by attempted serial transmission of their brain homogenates (**Fig. 1**). Neuropathological analysis of the brains of these mice excluded that neurologic dysfunction was caused by the spontaneous generation of prions. It is noteworthy that neurological deficits in Tg mice overexpressing PrP are not uncommon and are distinct from those caused by prion infection. Tg mice overexpressing wt MoPrP-B, Syrian hamster PrP, or ovine PrP develop disease featuring hindlimb paralysis, tremors, and ataxia, with mean ages of onset at ∼550 days [34]. Deletion of specific N-terminal segments of PrP results in fatal ataxia accompanied by degeneration of the cerebellum at 90–275 days of age [35]. Deletions of helical regions near the C-terminus result in CNS illnesses similar to neuronal storage diseases [36]. Like Tg9949 mice, none of these neurologically compromised mice spontaneously generated prions.

Evidence of prion disease was observed in 22 of 25 amyloid inoculations in Tg9949 mice, but was not observed from any of 7 control inoculations, including PBS, BSA, α-helical recPrP, β-oligomeric recPrP, and 3 uninfected Tg9949 brain homogenates. These results exclude the possibility that the observed neuropathology resulted from contamination of the inocula.

It is possible that a small titer of rPrP^Sc^ that eluded detection is responsible for the disease observed in these studies. Given the extensive neurodegeneration observed in the brains of infected Tg9949 mice (**Fig. S4**), this possibility seems unlikely. In fact, the vacuolation profile generated by inoculating the protease-resistant MoSP1 strain into Tg9949 mice was much less severe than that observed for MoSP2 prions, which lack protease-resistance [22]. Furthermore, despite its tendency to accumulate, no rPrP^Sc^ could be detected even upon serial passage (**Fig. 3**). Nonetheless, it is conceivable that some rPrP^Sc^ may be detectable under conditions not yet explored, for example, using alternate proteases. This would not alter our conclusions, however, that such protease-sensitive prions would be overlooked using the standard conditions used to detect prions.

Whereas protease-sensitive prions composed of mutant PrP^Sc^(P101L) in Tg mice have been studied extensively [15],[16],[17], wt sPrP^Sc^ has been less well investigated. While rPrP^Sc^ is clearly transmissible, it is unknown what role, if any, rPrP^Sc^ plays in the pathogenesis of prion disease. From the studies reported here as well as other investigations, sPrP^Sc^ is clearly pathogenic.

The pathogenicity of sPrP^Sc^ calls into question the adequacy of some terms used to describe different isoforms of PrP, such as PrP^res^ and PrP^sen^ [37]. PrP^res^ is often equated with PrP^Sc^, and PrP^sen^ with PrP^C^. From the work presented here, we contend that PrP^Sc^ can be both protease-resistant and protease-sensitive, rendering terms that describe only the protein's response to limited protease digestion as ambiguous. Therefore, the use of terms describing both infectivity and resistance to protease digestion (i.e., sPrP^Sc^, rPrP^Sc^, and PrP^C^) is necessary in order to avoid confusion.

While inoculation ic of recMoPrP(89–230) amyloid did not shorten the lives of Tg9949 mice (**Table S2**), the amyloid preparations provoked severe neurodegeneration (**Fig. 2**). Serial transmission of protease-sensitive prions MoSP2, MoSP3, and MoSP4 in Tg9949 mice did not alter the incubation periods (**Table S5**), suggesting that these prion isolates encipher long incubation times.

Because the formation of rPrP^Sc^ has been used as an operational assay for the identification of prions, protease resistance has been often viewed as an intrinsic and obligatory feature of prions [38]. The results reported here extend our more recent findings that challenge the notion that protease resistance is an obligatory feature of PrP^Sc^ that is required for the transmission of prions [4],[16].

The production of synthetic prions, which are sensitive to proteolysis but cause transmissible disease, is an important step toward understanding the role of protease-sensitive forms of PrP^Sc^ in the pathogenesis of prion disease. Recent reports suggest that prions with low levels of rPrP^Sc^ occur naturally in sheep [14] and humans [9]. Our results show the importance of using alternate methods for detecting PrP^Sc^, rather than employing only the presence of PK-resistant PrP. Exclusive reliance on the detection of rPrP^Sc^ as a surrogate marker for prion infectivity may overlook the contribution of sPrP^Sc^ to prion infectivity and the pathogenesis of prion disease [39].

## Materials and Methods

### Ethics statement

All animal procedures were performed under protocols approved by the Institutional Animal Care and Use Committee at the University of California San Francisco.

### Recombinant PrP

RecMoPrP(89–230) was made as previously described [22],[40]. For inoculation into Tg9949 mice, recMoPrP(89–230) was refolded into an α-helical conformation at 0.5 mg/ml [31], a β-rich oligomer at 1.0 mg/ml [32], or into amyloid fibers at 1.0 mg/ml [22]. For recPrP used in the ASA, lyophilized protein was dissolved in 6 M Gdn at 5 mg/ml, aliquotted, and stored at −80°C.

### Transgenic mice

Tg9949 mice [also referred to as Tg(MoPrP,Δ23–88)9949/*Prnp* ^0/0^ mice] were bred in-house and express MoPrP(89–231) on a knockout background at 16–32× compared to PrP in Syrian hamsters [21]. Tg4053 mice [also referred to as Tg(MoPrP-A)4053 mice] [28] were bred in-house and express full-length PrP at 4–8× the levels in wt, FVB mice [17]. FVB mice were obtained from Charles River Laboratories (Wilmington, MA).

### Preparation of brain homogenates

To prepare 10% (w/v) brain homogenates, 9 volumes of ice-cold PBS were added to brain tissue in a 50-ml tube. Brain tissue was homogenized on ice, using either needle extrusion through progressively smaller needles, or, for samples used in the ASA, by bead beating (FastPrep FP120, Qbiogene). The sample was centrifuged at 500× *g* for 5 min at room temperature (RT) to clarify samples. The supernatant was collected, the pellet discarded; aliquots were keep frozen at −80°C until use.

### Inoculation

RecPrP was inoculated following dialysis against PBS to remove toxic buffer components; alternatively, the fibers were washed 3× in PBS to remove toxic buffer components. Each time, fibrils were spun down at maximum speed in a tabletop centrifuge and resuspended in PBS as indicated in **Table S2**. For serial passage experiments, 10% brain homogenates from Tg9949 mice were diluted 1∶10 in 5% BSA in PBS. Approximately 30 µl of recPrP, PBS (with or without 5 mg/ml BSA), or diluted brain homogenate were inoculated intracerebrally into mice of either sex, aged 7 to 10 weeks. Inoculation was carried out with a 27-gauge, disposable hypodermic needle inserted into the right parietal lobe.

Mice were examined twice weekly for neurological dysfunction. Animals were assessed using standard diagnostic criteria for prion disease [41],[42]. If neurological dysfunction was evident, mice were sacrificed and their brains were removed for biochemical and histological analysis.

### PK digestion and Western blots

Brain homogenates were adjusted to 1 mg/ml total protein; 20 µg/ml PK (Boehringer Mannheim) was added for a final volume of 0.5 ml. Following a 1-h incubation at 37°C, digestion was stopped by addition of phenylmethylsulfonyl fluoride (PMSF; 2 mM final concentration). Digestion products were precipitated by centrifugation at 100,000× *g* for 1 h, resuspended in SDS loading buffer, and run on 12% polyacrylamide gels. Western blotting was carried out as previously described [41] using anti-PrP HuM-D18, P, or R2.

### Amyloid seeding assay

The ASA was performed as described elsewhere [26], except that PTA pellets were prepared on 1/5 scale (100 µl of 5% BH was used as starting material, and all volumes scaled down proportionally). Briefly, brain homogenates in Sarkosyl were precipitated with PTA to purify prions. Two µl of PTA-purified brain homogenates were diluted into 400 µl water, then tested as seeds in amyloid formation reactions. A 96-well plate was prepared with 180 µl/well of recPrP solution (50 µg/ml recMoPrP(89–230), 0.4 M GdnHCl, 1× PBS, 10 µM ThT). Twenty µl of diluted PTA-precipitated brain homogenate were added to each well, with each sample tested with six replicates. ThT fluorescence measurements were taken at 444/485 nm excitation/emission spectra on an M2 Spectramax fluorescence plate reader (Molecular Devices) after 6 h of continuous shaking at 37°C. Each sample was measured in six independent replicates.

### Prion strain MoSP1

MoSP1 used as a PK-resistant control in these experiments was passaged in either Tg9949 or Tg4053 mice [22].

### Neuropathology

Brains were fixed immediately upon being harvested by immersion in 10% buffered formalin. Following paraffin embedding, 8-µm-thick sections were stained with H&E to visualize vacuoles. Reactive astrocytic gliosis was visualized by peroxidase immunohistochemistry with an antibody against glial fibrillary acidic protein. The antibody R2 was used to visualize PrP deposits [43]. Distributions of neuropathological lesions were estimated as the percentage of tissue occupied by vacuoles. These estimates were confirmed by a second, independent technician.

### Statistical analysis

For survival analysis, STATA software (StataCorp, College Station, TX) was used to calculate p-values based on cumulative survival. Microsoft Excel (Microsoft Corp., Redmond, WA) was used to calculate standard deviations and standard errors.

## Supporting Information

Figure S1 Tg9949 mice with neurological dysfunction exhibit the same neuropathology associated with aging of wild-type and other transgenic mice. The cerebellum of a Tg9949 mouse exhibiting neurological dysfunction (ND) is compared with age-matched, healthy Tg9949 mice, wild-type FVB mice, and Tg4053 mice. Mild vacuolation (white holes observed in H&E-stained panels, top row) and astrocytic gliosis (dark brown spots labeled with anti-GFAP, bottom row) are observed in the white matter in all mice examined. Scale bar represents 100 µm and applies to all panels.
(0.31 MB PDF)

Figure S2 Sample kinetic data from the amyloid seeding assay. PTA pellets were generated from the brains of Tg9949 mice inoculated with BSA (blue diamonds), α-helical recPrP (blue triangles), β-oligomeric recPrP (blue circles), or amyloid fibrils of recPrP to generate MoSP2-1T (red triangles), MoSP3-1T (red circles), and MoSP4-1T (red squares); these pellets were added amyloid formation reactions in the presence of ThT. ThT fluorescence, indicating the presence of amyloid, was measured as a function of time. PTA pellets from MoSP2, MoSP3, and MoSP4 efficiently seeded amyloid formation, whereas the other PTA pellets did not. PTA pellets of uninoculated Tg9949 mice are also shown (blue squares).
(0.03 MB PDF)

Figure S3 Protease-resistant PrP is not detected in the brains of mice inoculated with MoSP2. Even at lower concentrations of PK, no difference in protease-resistant PrP fractions can be discerned between MoSP2-inoculated and uninoculated Tg9949 mice. Brain homogenates at protein concentrations of 1 mg/ml were incubated with PK at the indicated concentrations for 1 h at 37°C. The blot was probed with α-PrP antibody HuM-D18. Molecular weight standards are indicated on the left in kDa.
(0.02 MB PDF)

Figure S4 Vacuolation scores, estimated as the percentage of an area occupied by vacuoles, in different brain regions of Tg9949 (A) and Tg4053 mice (B) inoculated with MoSP2. (A) In Tg9949 mice, the first transmission (1T) and each subsequent serial transmission (2T and 3T) of MoSP2 resulted in widespread vacuolation, with comparable levels of vacuolation observed in each brain region. Note that no vacuolation (0%) is observed in BSA-inoculated Tg9949 mice. Asterisk indicates that age-related vacuolation was excluded in this scoring. Vacuolation resulting from passage of MoSP1 in Tg4053 mice (B) is shown for comparison. LC, limbic cortex; FC, frontal cortex; DG, dentate gyrus; CA, cornu ammonis of the hippocampus; LT, lateral thalamic nuclei; MT, medial thalamic nuclei; Cd, caudate nucleus; Cm, cerebellar molecular layer; Cg, cerebellar granule cell layer; Cw, cerebellar white matter; Bs, brainstem.
(0.04 MB PDF)

Figure S5 Histoblots of cerebellar brain sections show that PrP deposits in MoSP2-inoculated Tg9949 mice are protease sensitive. Sections were prepared from Tg9949 mice inoculated with brain homogenates of aged Tg9949 mice (control), MoSP1, or MoSP2. Only brains inoculated with MoSP1 show protease-resistant PrP. Histoblots from brains inoculated with MoSP2 are comparable to control Tg9949 mice. Histoblots were probed with HuM-D18.
(0.03 MB PDF)

Figure S6 Western blots of 5% Tg4053 brain homogenates after PK digestion and PTA precipitation reveal no rPrP^Sc^. PK digestion was performed at 20 µg/ml for 1 h at 37°C; PTA precipitation was performed in 2% Sarkosyl with 1% PTA at pH 7.4, for 1 h at 37°C. Brain homogenates from Tg4053 mice inoculated with either uninfected (-control) or MoSP1-infected Tg9949 brain homogenates are shown as controls. One ml of brain homogenate was precipitated, 30% of which was run on the gel, approximately 1000-fold as much homogenate as was used for the ASA. The blot was probed with µ-PrP antibody HuM-P. Apparent molecular masses based on the migration of protein standards are shown in kDa.
(0.02 MB PDF)

Table S1 Spontaneous neurological dysfunction in Tg9949 mice.
(0.04 MB PDF)

Table S2 Conditions used for the formation of amyloid fibers.
(0.02 MB PDF)

Table S3 Initial transmission of synthetic prions by inoculation of Tg9949 mice with amyloid fibers.
(0.02 MB PDF)

Table S4 Samples analyzed by ASA and neuropathology.
(0.03 MB PDF)

Table S5 Serial transmission of protease-sensitive synthetic prions in Tg9949 mice.
(0.01 MB PDF)

Table S6 Attempted serial transmission of MoSP2 prions to FVB mice.
(0.01 MB PDF)

Table S7 Attempted transmission of amyloid fibers to FVB mice.
(0.04 MB PDF)
