# Supplementary figures and images for: Protease-Sensitive Synthetic Prions
Source: PLoS Pathog. 2010 Jan 22;6(1):e1000736. doi: 10.1371/journal.ppat.1000736 (PMC2809756; doi:10.1371/journal.ppat.1000736)

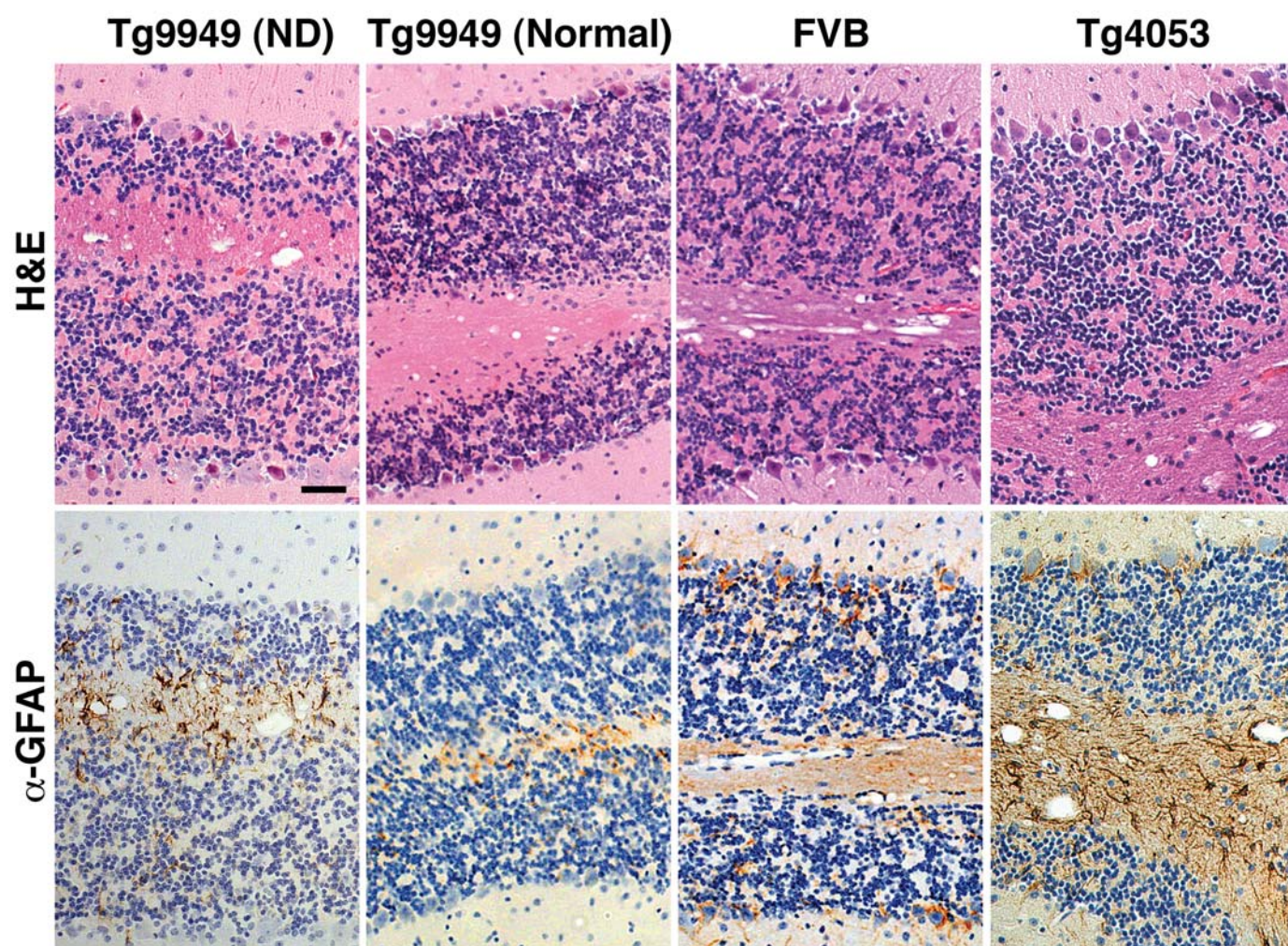

Supplement: Figure S1 — Tg9949 mice with neurological dysfunction exhibit the same neuropathology associated with aging of wild-type and other transgenic mice. The cerebellum of a Tg9949 mouse exhibiting neurological dysfunction (ND) is compared with age-matched, healthy Tg9949 mice, wild-type FVB mice, and Tg4053 mice. Mild vacuolation (white holes observed in H&E-stained panels, top row) and astrocytic gliosis (dark brown spots labeled with anti-GFAP, bottom row) are observed in the white matter in all mice examined. Scale bar represents 100 µm and applies to all panels. (0.31 MB PDF) [file ppat.1000736.s001.pdf]

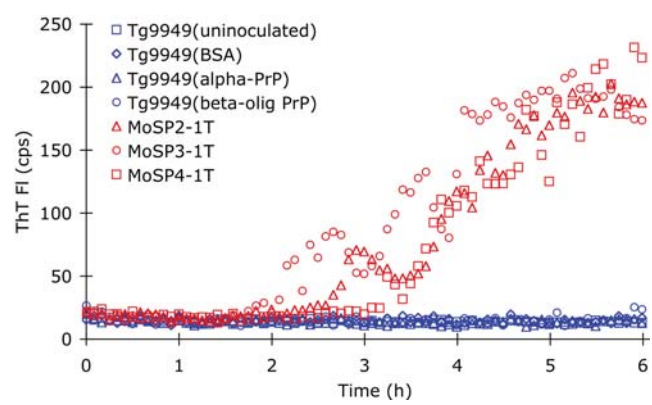

Supplement: Figure S2 — Sample kinetic data from the amyloid seeding assay. PTA pellets were generated from the brains of Tg9949 mice inoculated with BSA (blue diamonds), α-helical recPrP (blue triangles), β-oligomeric recPrP (blue circles), or amyloid fibrils of recPrP to generate MoSP2-1T (red triangles), MoSP3-1T (red circles), and MoSP4-1T (red squares); these pellets were added amyloid formation reactions in the presence of ThT. ThT fluorescence, indicating the presence of amyloid, was measured as a function of time. PTA pellets from MoSP2, MoSP3, and MoSP4 efficiently seeded amyloid formation, whereas the other PTA pellets did not. PTA pellets of uninoculated Tg9949 mice are also shown (blue squares). (0.03 MB PDF) [file ppat.1000736.s002.pdf]

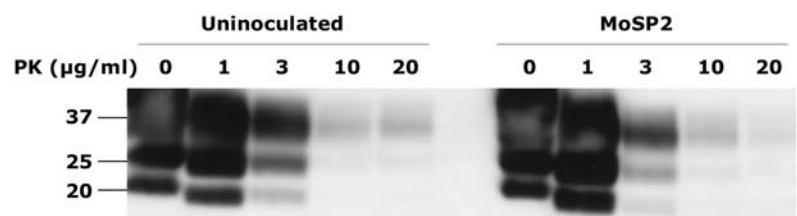

Supplement: Figure S3 — Protease-resistant PrP is not detected in the brains of mice inoculated with MoSP2. Even at lower concentrations of PK, no difference in protease-resistant PrP fractions can be discerned between MoSP2-inoculated and uninoculated Tg9949 mice. Brain homogenates at protein concentrations of 1 mg/ml were incubated with PK at the indicated concentrations for 1 h at 37°C. The blot was probed with α-PrP antibody HuM-D18. Molecular weight standards are indicated on the left in kDa. (0.02 MB PDF) [file ppat.1000736.s003.pdf]

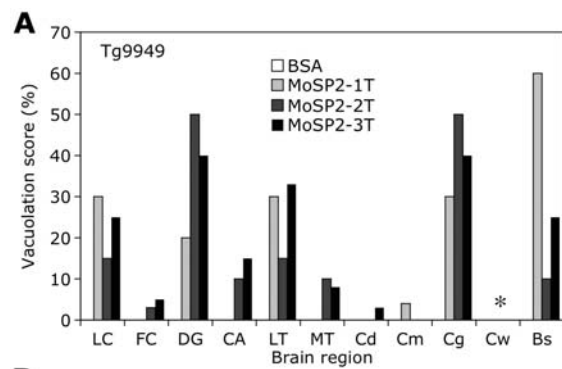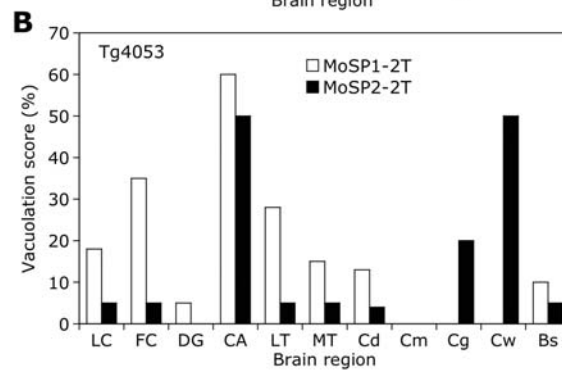

Supplement: Figure S4 — Vacuolation scores, estimated as the percentage of an area occupied by vacuoles, in different brain regions of Tg9949 (A) and Tg4053 mice (B) inoculated with MoSP2. (A) In Tg9949 mice, the first transmission (1T) and each subsequent serial transmission (2T and 3T) of MoSP2 resulted in widespread vacuolation, with comparable levels of vacuolation observed in each brain region. Note that no vacuolation (0%) is observed in BSA-inoculated Tg9949 mice. Asterisk indicates that age-related vacuolation was excluded in this scoring. Vacuolation resulting from passage of MoSP1 in Tg4053 mice (B) is shown for comparison. LC, limbic cortex; FC, frontal cortex; DG, dentate gyrus; CA, cornu ammonis of the hippocampus; LT, lateral thalamic nuclei; MT, medial thalamic nuclei; Cd, caudate nucleus; Cm, cerebellar molecular layer; Cg, cerebellar granule cell layer; Cw, cerebellar white matter; Bs, brainstem. (0.04 MB PDF) [file ppat.1000736.s004.pdf]

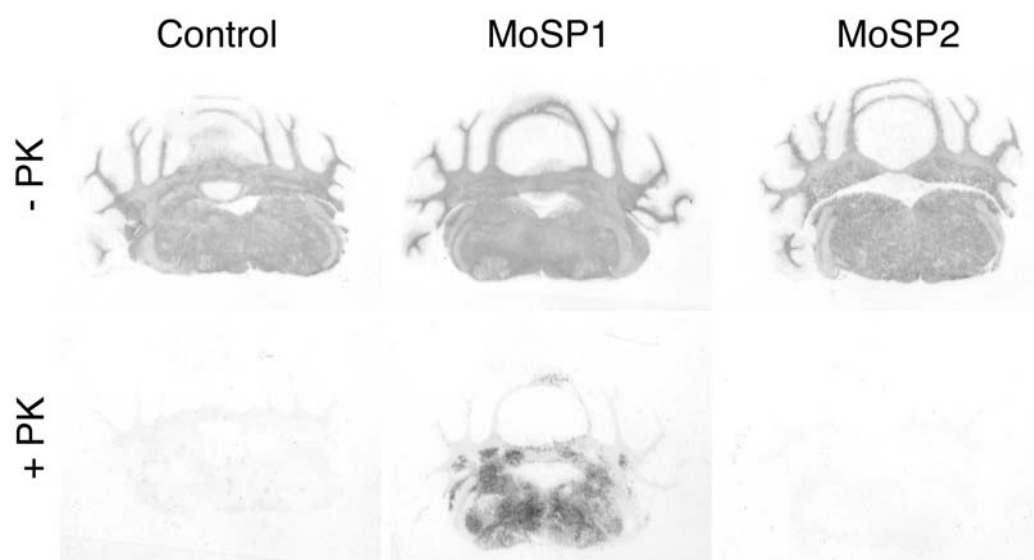

Supplement: Figure S5 — Histoblots of cerebellar brain sections show that PrP deposits in MoSP2-inoculated Tg9949 mice are protease sensitive. Sections were prepared from Tg9949 mice inoculated with brain homogenates of aged Tg9949 mice (control), MoSP1, or MoSP2. Only brains inoculated with MoSP1 show protease-resistant PrP. Histoblots from brains inoculated with MoSP2 are comparable to control Tg9949 mice. Histoblots were probed with HuM-D18. (0.03 MB PDF) [file ppat.1000736.s005.pdf]

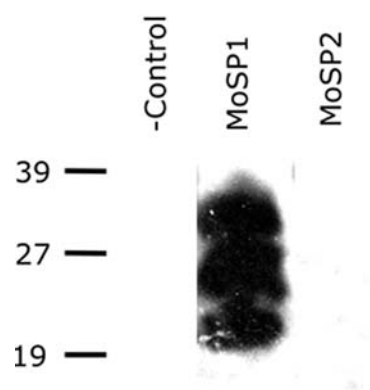

Supplement: Figure S6 — Western blots of 5% Tg4053 brain homogenates after PK digestion and PTA precipitation reveal no rPrPSc. PK digestion was performed at 20 µg/ml for 1 h at 37°C; PTA precipitation was performed in 2% Sarkosyl with 1% PTA at pH 7.4, for 1 h at 37°C. Brain homogenates from Tg4053 mice inoculated with either uninfected (-control) or MoSP1-infected Tg9949 brain homogenates are shown as controls. One ml of brain homogenate was precipitated, 30% of which was run on the gel, approximately 1000-fold as much homogenate as was used for the ASA. The blot was probed with µ-PrP antibody HuM-P. Apparent molecular masses based on the migration of protein standards are shown in kDa. (0.02 MB PDF) [file ppat.1000736.s006.pdf]
