# Supplementary material for: Protease-Sensitive Synthetic Prions
Source: PLoS Pathog. 2010 Jan 22;6(1):e1000736. doi: 10.1371/journal.ppat.1000736 (PMC2809756; doi:10.1371/journal.ppat.1000736)
Supplement: Table S1 — Spontaneous neurological dysfunction in Tg9949 mice. (0.04 MB PDF) [file ppat.1000736.s007.pdf]

Table S1. Spontaneous neurological dysfunction in Tg9949 mice.[a]

Host	Inoculum	n/n_0	Age of onset ± S.E. (days)	PK resistance	ASA activity	Neuropathology	Prion disease incidence (%)
FVB	PBS/BSA	2/12	512 ± 53	0/2	0/2	0/2	0
Tg4053	PBS/BSA	12/62	627 ± 39	0/3	0/3	0/2	0
Tg9949	None	62/96	617 ± 6.5	0/62	0/3	0/10	0
Tg9949	PBS/BSA	51/78	597 ± 8.0	0/51	0/6	0/10	0

[a] *Mice were inoculated at 7–10 weeks of age;* n, *number of mice with neurological dysfunction;* n_0, *number of inoculated mice. For PK resistance, ASA activity, and neuropathology, the number of positive samples over the number of samples examined is reported. Mice not exhibiting ataxia died of other causes, such as tumors, respiratory or skin diseases.*
