# Supplementary material for: Protease-Sensitive Synthetic Prions
Source: PLoS Pathog. 2010 Jan 22;6(1):e1000736. doi: 10.1371/journal.ppat.1000736 (PMC2809756; doi:10.1371/journal.ppat.1000736)
Supplement: Table S2 — Conditions used for the formation of amyloid fibers. (0.02 MB PDF) [file ppat.1000736.s008.pdf]

Table S2. Conditions used for the formation of amyloid fibers.

Amyloid fiber prep ID[a]	Starting PrP conformation	Denaturant Urea/Gdn (M)	Times seeded	Freeze-thaw seed	Freeze-thaw inoculum	Fiber purification method
1[b]	α-helical	4 U	1	–	–	Spun
2[b]	α-helical	4 U	0	–	–	Spun
3	α-helical	4 U	1	–	–	Spun
4	α-helical	4 U	0	–	–	Spun
14	α-helical	3 U	1	–	–	Dialyzed
15	α-helical	3 U	1	–	+	Dialyzed
16	α-helical	3 U	1	+	–	Dialyzed
17	α-helical	3 U	1	+	+	Dialyzed
18	α-helical	3 U	5	+	–	Dialyzed
19	α-helical	3 U	5	+	+	Dialyzed
20	α-helical	3 U	10	+	–	Dialyzed
21	α-helical	3 U	10	+	+	Dialyzed
22	α-helical	4 U	1	–	–	Dialyzed
23	α-helical	4 U	1	–	+	Dialyzed
24	α-helical	4 U	1	+	–	Dialyzed
25	α-helical	4 U	1	+	+	Dialyzed
26	α-helical	4 U	5	+	–	Dialyzed
27	α-helical	4 U	5	+	+	Dialyzed
28	α-helical	4 U	10	+	–	Dialyzed
29	α-helical	4 U	10	+	+	Dialyzed
30	β-rich	3 U	1	–	–	Dialyzed
31	β-rich	3 U	1	–	–	Spun
32	β-rich	3 U	10	–	–	Dialyzed
33	β-rich	3 U	10	–	–	Spun
34	α-helical	3 U + 1.2 G	1	–	–	Spun
35	α-helical	3 U + 1.2 G	1	–	–	Dialyzed

[a]*Amyloid preparations 5–13 are published in [1].*

[b]*Amyloids 1 and 2 were described in [2].*

References:

1. Colby DW, Giles K, Legname G, Wille H, Baskakov IV, et al. (2009) Design and construction of diverse mammalian prion strains. Proc Natl Acad Sci USA 106: 20417–20422.

2. Legname G, Baskakov IV, Nguyen H-OB, Riesner D, Cohen FE, et al. (2004) Synthetic mammalian prions. Science 305: 673-676.
