# Supplementary material for: Protease-Sensitive Synthetic Prions
Source: PLoS Pathog. 2010 Jan 22;6(1):e1000736. doi: 10.1371/journal.ppat.1000736 (PMC2809756; doi:10.1371/journal.ppat.1000736)
Supplement: Table S3 — Initial transmission of synthetic prions by inoculation of Tg9949 mice with amyloid fibers. (0.02 MB PDF) [file ppat.1000736.s009.pdf]

Table S3. Initial transmission of synthetic prions by inoculation of Tg9949 mice with amyloid fibers. [a]

**Inoculum**[b]	***n***	**Median age (days)**	**PK resistance**	**ASA activity**	**Neuro-pathology**	**Prion disease incidence (%)**	**Resulting prion isolate**
recPrP(89–230) α-helical monomer	10	620	0/2	0/2	0/2	0	None
recPrP(89–230) β-rich oligomer	8	574	0/2	0/2	0/2	0	None
Amyloid fibers 1[b]	8	567	5/5	n.d.	2/2	100	MoSP1
Amyloid fibers 2[b]	8	591	0/3	2/2	2/2	100	MoSP2-1T
Amyloid fibers 3	10	611	0/3	3/3	2/2	100	MoSP3-1T
Amyloid fibers 4	10	573	0/7	3/3	2/2	100	MoSP4-1T
Amyloid fibers 14	8	548	0/4	3/4	1/1	75	n.a.
Amyloid fibers 15	8	614	0/4	4/4	2/2	100	n.a.
Amyloid fibers 16	8	669	0/3	3/3	2/2	100	n.a.
Amyloid fibers 17	8	645	0/2	2/2	1/1	100	n.a.
Amyloid fibers 18	8	496	0/6	3/4	1/3	75	n.a.
Amyloid fibers 19	8	570	0/3	3/3	2/2	100	MoSP19
Amyloid fibers 20	8	639	0/3	3/3	3/3	100	n.a.
Amyloid fibers 21	8	665	0/6	0/6	0/2	0	None
Amyloid fibers 22	8	619	0/4	3/4	2/3	75	n.a.
Amyloid fibers 23	8	579	0/4	3/6	2/4	50	n.a.
Amyloid fibers 24	8	577	0/7	0/7	0/3	0	None
Amyloid fibers 25	8	622	0/3	3/3	3/3	100	n.a.
Amyloid fibers 26	8	636	0/5	0/5	0/3	0	None
Amyloid fibers 27	8	625	0/6	4/6	n.d.	67	n.a.
Amyloid fibers 28	8	589	0/2	2/2	2/2	100	n.a.
Amyloid fibers 29	8	620	0/2	2/2	2/2	100	n.a.
Amyloid fibers 30	8	619	0/2	2/2	2/2	100	n.a.
Amyloid fibers 31	8	652	0/2	3/3	3/3	100	n.a.
Amyloid fibers 32	8	650	0/2	3/3	2/2	100	n.a.
Amyloid fibers 33	8	646	0/2	2/3	1/1	67	n.a.
Amyloid fibers 34	8	639	0/2	4/4	2/2	100	n.a.
Amyloid fibers 35	8	625	0/2	2/2	2/2	100	n.a.

[a] *Mice were inoculated at 7–10 weeks of age;* n, *number of inoculated mice. For PK resistance, ASA activity, and neuropathology, the number of positive samples over the number of samples examined is reported. Prion disease incidence is calculated based on ASA results, which correlate with neuropathology for 98% of samples.*

[b] *Amyloid preparations 5–13 are published in [1]. Initial transmission of MoSP1 and MoSP2 by inoculation of Tg9949 mice with amyloid fibers composed of recPrP(89–230) was described in [2].*

n.d., not determined; n.a., not applicable; only prion isolates used for further study were named.

References:

1. Colby DW, Giles K, Legname G, Wille H, Baskakov IV, et al. (2009) Design and construction of diverse mammalian prion strains. Proc Natl Acad Sci USA 106: 20417–20422.

2. Legname G, Baskakov IV, Nguyen H-OB, Riesner D, Cohen FE, et al. (2004) Synthetic mammalian prions. Science 305: 673-676.
