# Supplementary material for: Protease-Sensitive Synthetic Prions
Source: PLoS Pathog. 2010 Jan 22;6(1):e1000736. doi: 10.1371/journal.ppat.1000736 (PMC2809756; doi:10.1371/journal.ppat.1000736)
Supplement: Table S4 — Samples analyzed by ASA and neuropathology. (0.03 MB PDF) [file ppat.1000736.s010.pdf]

Table S4. Samples analyzed by ASA and neuropathology.

Inoculum	Animal ID#	ASA	Neuropathology
Amyloid fibers 14	MN18048	+	+
Amyloid fibers 15	MN18068	+	+
	MN18071	+	+
Amyloid fibers 16	MN18092	+	+
	MN18094	+	+
Amyloid fibers 17	MN18714	+	+
Amyloid fibers 18	MN18724	-	-
	MN18716	+	-
	MN18721	+	+
Amyloid fibers 19	MN18742	+	+
	MN21523	+	+
Amyloid fibers 20	MN21518	+	+
	MN21524	+	+
	MN21526	+	+
Amyloid fibers 21	MN21539	-	-
	MN21540	-	-
Amyloid fibers 22	MO34	+	+
Amyloid fibers 23	MO37	+	+
	MO39	+	+
	MO46	-	-
	MO51	-	-
Amyloid fibers 24	MO57	-	-
	MO59	-	-
	MO60	-	-
Amyloid fibers 25	MO72	+	+
	MO75	+	+
	MO70	+	+
Amyloid fibers 26	MO79	-	-
	MO82	-	-
	MO468	-	-
Amyloid fibers 28	MO1019	+	+
	MO1028	+	+
Amyloid fibers 29	MO1032	+	+
	MO1037	+	+
Amyloid fibers 30	MO10925	+	+
	MO10931	+	+
Amyloid fibers 31	MO9112	+	+
	MO9101	+	+
	MO11199	+	+
Amyloid fibers 32	MO11202	+	+
	MO11206	+	+
Amyloid fibers 33	MO12155	+	+
Amyloid fibers 34	MO10903	+	+
	MO10905	+	+
Amyloid fibers 35	MO11164	+	+
	MO11166	+	+
