# Supplementary material for: Protease-Sensitive Synthetic Prions
Source: PLoS Pathog. 2010 Jan 22;6(1):e1000736. doi: 10.1371/journal.ppat.1000736 (PMC2809756; doi:10.1371/journal.ppat.1000736)
Supplement: Table S5 — Serial transmission of protease-sensitive synthetic prions in Tg9949 mice. (0.01 MB PDF) [file ppat.1000736.s011.pdf]

Table S5. Serial transmission of protease-sensitive synthetic prions in Tg9949 mice.[a]

**Inoculum**	***n***	**Age ± S.E. (days)**	**PK resistance**	**ASA activity**	**Neuro-pathology**	**Prion disease incidence (%)**	**Resulting prion isolate**
None	62	617 ± 6.5	0/62	0/3	0/10	0	None
Ataxic Tg9949 Control A	7	618 ± 26	0/2	0/2	0/2	0	None
Ataxic Tg9949 Control B	6	634 ± 49	0/2	0/2	0/2	0	None
Ataxic Tg9949 Control C	4	650 ± 23	0/2	0/2	0/2	0	None
MoSP2-1T	25	598 ± 13	0/2	3/3	2/2	100	MoSP2-2T
MoSP2-2T	32	584 ± 9.7	0/2	3/3	2/2	100	MoSP2-3T
MoSP3-1T	12	589 ± 11	0/2	3/3	2/2	100	MoSP3-2T
MoSP4-1T	15	559 ±12	0/2	3/3	2/2	100	MoSP4-2T

[a] *Inocula were prepared from brain homogenates of either ataxic Tg9949 mice or Tg9949 mice containing the prion isolate indicated. Mice were inoculated at 7–10 weeks of age;* n*, number of ataxic/ill mice in study. For PK resistance, ASA activity, and neuropathology, the number of positive samples over the number of samples examined is reported.*
