# Supplementary material for: Protease-Sensitive Synthetic Prions
Source: PLoS Pathog. 2010 Jan 22;6(1):e1000736. doi: 10.1371/journal.ppat.1000736 (PMC2809756; doi:10.1371/journal.ppat.1000736)
Supplement: Table S6 — Attempted serial transmission of MoSP2 prions to FVB mice. (0.01 MB PDF) [file ppat.1000736.s012.pdf]

Table S6. Attempted serial transmission of MoSP2 prions to FVB mice.[a]

Inoculum	n/n_0	Age ± S.E. (days)	PK resistance	ASA activity	Neuropathology	Prion disease incidence (%)
PBS/BSA	2/12	452 ± 53	0/2	0/2	0/2	0
MoSP2-1T	3/10[b]	675 ± 25	0/3	0/3	0/2	0

[a] *Inoculum was prepared from the brain homogenate of Tg9949 mice containing MoSP2. Control mice were inoculated with BSA.* n, *number of ill mice;* n_0 *number of inoculated mice. For PK resistance, ASA activity, and neuropathology, the number of positive samples over the number of samples examined is reported.*

[b] *Three FVB mice inoculated with MoSP2 exhibited signs of ataxia, but biochemical and neuropathological analysis revealed no signs of prion disease. The occurrence of ataxia in these mice was consistent with BSA-inoculated, control mice.*
