# Supplementary material for: Protease-Sensitive Synthetic Prions
Source: PLoS Pathog. 2010 Jan 22;6(1):e1000736. doi: 10.1371/journal.ppat.1000736 (PMC2809756; doi:10.1371/journal.ppat.1000736)
Supplement: Table S7 — Attempted transmission of amyloid fibers to FVB mice. (0.04 MB PDF) [file ppat.1000736.s013.pdf]

Table S7. Attempted transmission of amyloid fibers to FVB mice.[a]

Inoculum	n/n_0	Mean incubation period (days ± SEM)	p-value
Amyloid fibers 14	0/8	n.a.	> 0.30
Amyloid fibers 15	4/8	506 ± 46	0.14
Amyloid fibers 16	0/8	n.a.	> 0.30
Amyloid fibers 17	0/8	n.a.	> 0.30
Amyloid fibers 18	0/8	n.a.	> 0.30
Amyloid fibers 19	0/8	n.a.	> 0.30
Amyloid fibers 20	0/8	n.a.	> 0.30
Amyloid fibers 21	1/8	694	> 0.30
Amyloid fibers 22	1/8	543	> 0.30
Amyloid fibers 23	1/8	567	> 0.30
Amyloid fibers 24	2/8	650 ± 44	> 0.30
Amyloid fibers 25	0/8	n.a.	> 0.30
Amyloid fibers 26	2/8	533 ± 144	> 0.30
Amyloid fibers 27	2/8	685 ± 36	> 0.30
Amyloid fibers 28	1/8	539	> 0.30
Amyloid fibers 29	1/8	455	> 0.30

[a] *Mice were inoculated at 7–10 weeks of age; n, number of ill mice; n_0, number of inoculated mice. For each inoculation, mice were observed until all animals died of natural causes, exhibited neurological dysfunction, or lived for >850 d. For the cumulative incidence of neurological dysfunction, only Amyloid fibers 15 yielded a potential difference compared to BSA inoculation (p = 0.14). However, Western blot analysis and ASA using brain tissues from the mice in this experiment did not indicate the presence of prions.*
